# CPL2 and CPL3 act redundantly in *FLC* activation and flowering time regulation in *Arabidopsis*

**DOI:** 10.1080/15592324.2022.2026614

**Published:** 2022-02-03

**Authors:** Yu Zhang, Lisha Shen

**Affiliations:** Temasek Life Sciences Laboratory, National University of Singapore, Singapore, Singapore

**Keywords:** Flowering time, FLC, CPL2, CPL3, Arabidopsis

## Abstract

Reproductive success of plants greatly depends on the proper timing of the floral transition, which is precisely controlled by a complex genetic network. *FLOWERING LOCUS C* (*FLC*), a central floral repressor, is transcriptionally activated by the FRIGIDA (FRI) activator complex including FLC EXPRESSOR (FLX) and FLX-LIKE 4 (FLX4). C-TERMINAL DOMAIN PHOSPHATASE-LIKE 3 (CPL3) forms a protein complex with FLX and FLX4 to mediate the dephosphorylation of FLX4, thereby promoting *FLC* expression to repress flowering in both winter and summer annuals. Here, we show that CPL2 acts redundantly with CPL3 to mediate *FLC* activation and flowering time. Similar to CPL3, CPL2 inhibits the floral transition, and is required for basal *FLC* expression in summer annuals and *FLC* activation in winter annuals. CPL2 directly interacts with FLX which further bridges the interaction between CPL2 and FLX4. Our results suggest that CPL2 and CPL3 function redundantly in regulating *FLC* expression to prevent precocious flowering.

The transition from vegetative development to reproductive development in flowering plants, known as the floral transition, is precisely controlled to occur at the appropriate time for their reproductive success. In the model plant *Arabidopsis thaliana*, multiple flowering genetic pathways including the photoperiod, autonomous, vernalization, thermosensory, gibberellin, and aging pathways, integrate different environmental and developmental flowering signals to ensure the proper timing of the floral transition.^1–3^ Among these pathways, the vernalization pathway senses a prolong period of winter cold exposure to repress the expression of a key flowering repressor, *FLOWERING LOCUS C* (*FLC*) to induce flowering.^4^ Based on whether vernalization is needed for rapid flowering, *Arabidopsis* accessions can be classified into two groups: winter annuals and summer annuals. In winter annuals, *FLC* expression is activated by the FRIGIDA (FRI) activator complex (FRI-C) consisting of FRI, FLC EXPRESSOR (FLX), FLX-LIKE 4 (FLX4), SUPPRESSOR OF FRI 4 (SUF4), FRI ESSENTIAL 1 (FES1), and FRI-LIKE 1 (FRL1).^5–8^ In contrast, basal levels of *FLC* expression is established by several regulators including FLX and FLX4 in rapid-cycling summer annuals like Columbia (Col) that contains a nonfunctional *fri* allele.^6^

We have recently shown that activation of *FLC* expression in flowering time control requires the RNA polymerase II (Pol II) C-terminal domain phosphatase-like protein, C-TERMINAL DOMAIN PHOSPHATASE-LIKE 3 (CPL3),^9^ which has also been shown to mediate the dephosphorylation of RNA Pol II to regulate plant immune response.^10,11^ CPL3 physically interacts with and dephosphorylates FLX4 through their common interacting protein FLX to enable the binding of dephosphorylated FLX4 to the *FLC* locus, thereby promoting *FLC* expression to inhibit flowering in both winter and summer annuals. In winter annuals, disruption of either *CPL3, FLX* or *FLX4* could greatly suppress the extremely late-flowering phenotype of *FRI*.^6,8,9^ However, unlike *flx* or *flx4* that fully suppresses the *FRI* phenotype, *cpl3* mutant could not completely suppress the late flowering of *FRI*,^9^ implying that other regulator(s) may act redundantly with CPL3 in mediating FRI-dependent *FLC* activation.

In the *Arabidopsis* genome, there are several other CTD phosphatase-like proteins, including CPL1/FIERY2, CPL2 and CPL4,^12,13^ which potentially function redundantly with CPL3 in flowering time control. Like CPL3, CPL4 contains a catalytic domain and a BRCA1 C Terminus (BRCT) domain at its C-terminus, whereas CPL1 and CPL2 each contain a catalytic domain and 1–2 dsRNA-binding domains (Figure 1(a)). We examined the potential redundancy between CPL3 and these three CPL proteins by examining the flowering phenotype of their mutants or their interaction with FLX or FLX4. Yeast two-hybrid assay revealed that CPL4 did not interact with CPL3 or components in FRI-C including FLX, FLX4, SUF4, and FES1 (Figure 1(b)), excluding the possibility of CPL4 as a redundant factor of CPL3. Moreover, *CPL4* is essential for plant viability and knockdown of *CPL4* results in strong growth defects in plant growth,^14,15^ preventing us from examining the flowering phenotype of *cpl4* mutants. To examine the effect of CPL1 and CPL2 on flowering time control, we obtained seeds of *cpl1-8* (GK-165H09-013365)^16^ and *cpl2-2* (SALK_059753),^13^ from the *Arabidopsis* Biological Resource Center. *cpl1-8* mutants exhibited a clear late-flowering phenotype under long days (Figure 1(c)) similar to *cpl1-3*,^17^ and elevated *FLC* expression during the floral transition (Figure 1(d)), implying that CPL1 and CPL3 have distinct functions in flowering time regulation.

**Figure 1.**
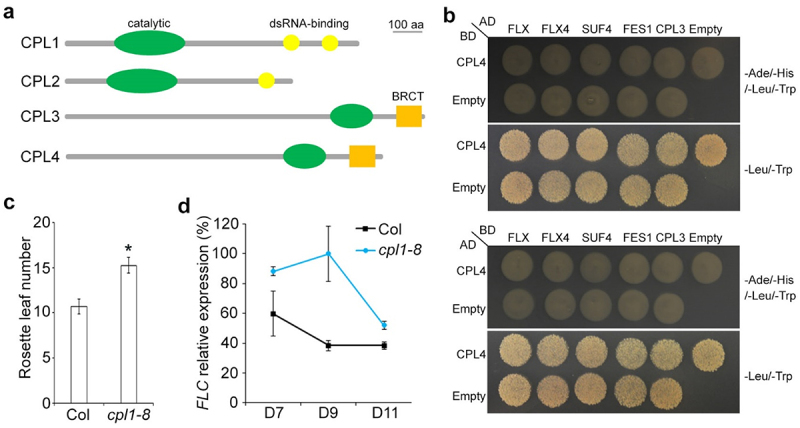
CPL proteins in *Arabidopsis*. (a) Schematic diagrams showing the domain structures of CPL proteins. (b) CPL4 does not interact with FLX, FLX4, SUF4, FES1 or CPL3. Transformed yeast cells were grown on SD-Ade/His/-Leu/-Trp medium and SD-Leu/-Trp medium. (c) *cpl1* mutants show late flowering under long days. Error bars, mean ± s.d.; n = 15. The asterisk denotes a significant difference in the flowering time between *cpl1-8* and wild-type (Col) (two-tailed paired Student’s *t* test, *P* < .05). (d) Temporal expression of *FLC* in developing seedlings of wild-type (Col) and *cpl1* mutants under long days. The levels of gene expression normalized to *TUB2* expression are shown relative to the maximal expression level set at 100%. Error bars, mean ± s.d.; n = 3 biological replicates.

Interestingly, *cpl2-2* showed accelerated flowering under long days (Figure 2(a)), which has been consistently shown in a previous study.^13^ The early-flowering phenotypes of *cpl2-2* prompted us to investigate whether it functions redundantly with CPL3 to regulate *FLC* activation. To this end, we first examined the expression levels of *FLC* in *cpl2-2* mutants and found that *FLC* expression was significantly downregulated in *cpl2-2* mutants (Figure 2(b)). *FLC* expression was consistently downregulated in the developing seedlings of *cpl2-2* before, during and after the floral transition (Figure 2(c)). Next, we examined whether CPL2 is required for *FRI*-dependent *FLC* activation in winter annuals. We crossed *cpl2-2* with *FRI*-Col,^18^ and found that *cpl2-2* partially suppressed the extremely late-flowering phenotype of *FRI* (Figure 2(a)). Consistently, further expression analysis showed that *FLC* expression was reduced in *FRI cpl2-2* as compared with *FRI* (Figure 2(b)). These observations indicate that CPL2 promotes *FLC* expression to inhibit flowering in both summer annuals and winter annuals, and that CPL2 may act redundantly with CPL3 in *FLC* activation.

**Figure 2.**
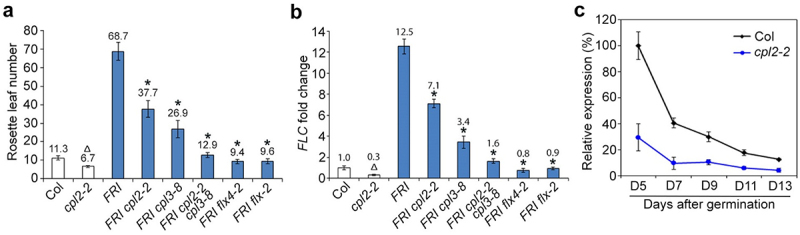
CPL2 acts redundantly with CPL3 in regulating *FLC*. (a) *cpl2-2* shows early flowering and *cpl2-2 cpl3-8* almost completely suppresses the late-flowering phenotype of *FRI* under long days. Error bars, mean ± s.d.; n = 20. A triangle denotes a significant difference in flowering time between *cpl2-2* and wild-type (Col), while asterisks denote significant differences in flowering time between indicated genotypes and *FRI* (two-tailed paired Student’s *t* test, *P* < .05). (b) *FLC* expression in 9-day-old seedlings of various genotypes determined by quantitative real-time PCR. *FLC* expression in wild-type (Col) was set as 1.0. Error bars, mean ± s.d.; n = 3 biological replicates. A triangle denotes significant differences in *FLC* expression between *cpl2-2* and wild-type (Col), while asterisks denote significant differences in *FLC* expression between indicated genotypes and *FRI* (two-tailed paired Student’s *t* test, *P* < .05). (c) Temporal expression of *FLC* determined by quantitative real-time PCR in developing seedlings of wild-type (Col) and *cpl2-2*. The levels of gene expression normalized to *TUB2* expression are shown relative to the maximal expression level set at 100%. Error bars, mean ± s.d.; n = 3 biological replicates.

CPL3 forms a protein complex with FLX and FLX4.^9^ Similar to CPL3, CPL2 interacted with FLX but not FLX4 in yeast two-hybrid assays (Figure 3(a)). Further yeast three-hybrid assay revealed that CPL2 interacted with FLX4 in the presence of FLX (Figure 3(b)). These results suggest that CPL2 interacts with FLX4 through their scaffold protein FLX like CPL3 does. Interestingly, we found that CPL2 also interacted with CPL3 in yeast (Figure 3(c)), indicating that these two proteins may form heterodimers.

**Figure 3.**
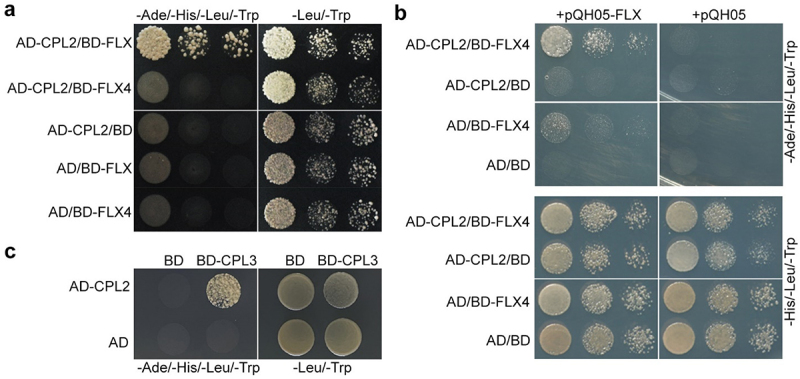
CPL2 interacts with FLX4 in the presence of FLX. (a) Yeast two-hybrid results showing the interaction between CPL2 and FLX. Serial dilution (1:1, 1:5, 1:10) of transformed yeast cells were grown on SD-Ade/-His/-Leu/-Trp (left panel) and SD-Leu/-Trp (right panel). (b) Indirect interaction between CPL2 and FLX4 via FLX. Serial dilution (1:1, 1:5, 1:10) of transformed yeast cells were grown on SD-Ade/-His/-Leu/-Trp (upper panel) and SD-His/-Leu/-Trp (lower panel). (c) Yeast two-hybrid results showing the interaction between CPL2 and CPL3. Transformed yeast cells were grown on SD-Ade/-His/-Leu/-Trp (left panel) and SD-Leu/-Trp (right panel).

Next, we crossed *FRI cpl2-2* with *cpl3-8* to generate the *FRI cpl2-2 cpl3-8* triple mutants. Either *cpl2-2* or *cpl3-8* partially suppressed the extremely late-flowering phenotype of *FRI*, whereas mutations in both *CPL2* and *CPL3* almost completely suppressed the late-flowering phenotype of *FRI* (Figure 2(a)). Consistently, *FLC* expression was almost fully restored in *FRI cpl2-2 cpl3-8* triple mutants to the levels in Col (Figure 2(b)). However, *FLC* expression was slightly higher in *FRI cpl2-2 cpl3-8* than *FRI flx4-2* or *FRI flx-2* (Figure 2(b)), indicating the possible involvement of other regulator(s) functioning redundantly or in parallel with CPL2 and CPL3 in regulating *FLC* expression.

Overall, our results suggest that CPL2 acts redundantly with CPL3 in *FLC* activation and flowering time regulation. Both CPL2 and CPL3 inhibit flowering in *Arabidopsis* and are required for the basal *FLC* expression in summer annuals and FRI-dependent *FLC* upregulation in the winter annuals. Although either *cpl2* or *cpl3* mutants only partially suppress *FRI* flowering phenotype, *cpl2 cpl3* double mutants almost fully suppress the late flowering of *FRI*. CPL2/3 forms a protein complex with FLX and FLX4, two components in the FRI-C transcriptional activator complex. Since CPL3 has been shown to dephosphorylate FLX4 protein^9^ and CPL2 also possesses the phosphatase activity,^10^ it is possible that CPL2 also modulates the phosphorylation status of FLX4 to regulate *FLC* expression, thus affecting flowering. Besides CPL2/3, there are many other CTD phosphatases in *Arabidopsis*,^19^ our revealed CPL2/3-FLX-FLX4-*FLC* functional module sheds new lights on the biological roles of CTD phosphatases in mediating dephosphorylation of key developmental regulators to modulate gene expression in plants.

## Materials and methods

### Plant materials and growth conditions

Seeds of *Arabidopsis* were placed on soil and stratified at 4°C in darkness for 3 days before they were grown under long days (16 hr light/8 hr dark) at 23 ± 2°C. Mutant seeds of *cpl2-2* (SALK_059753) and *cpl1-8* (GK-165H09-013365) were obtained from the *Arabidopsis* Information Resource, while seeds of *flx-2* and *FRI flx4-2* were kindly provided by Prof. Scott Michaels (Indiana University).

### Expression analysis

Total RNA was extracted using the RNeasy Plus Mini Kit (Qiagen) and reverse transcribed with the M-MLV Reverse Transcriptase (Promega) following manufacturers’ instructions. Quantitative real-time PCR was performed using SYBR Green PCR Master Mix (Applied Biosystems) with three independently collected biological samples. The expression of *TUBULLIN* 2 (*TUB2*) was used as the internal control. The normalized expression of target genes is calculated with the difference between the cycle threshold (Ct) of target genes and the Ct of control.

### Yeast two-hybrid and three-hybrid assays

The coding sequences of *CPL2, CPL3, SUF4, FES1, FLX4* and *FLX* were cloned into pGBKT7 (BD) or pGADT7 (AD) vectors (Clontech). For yeast two-hybrid assay, various combinations of AD and BD vectors were co-transformed into Y2HGold yeast cells using the Yeastmaker Yeast Transformation System 2 according to the manufacturer’s instructions (Clontech). After transformation, yeast cells were grown on SD-Leu/-Trp and SD-Ade/-His/-Leu/-Trp mediums. For yeast three-hybrid assay, the coding sequence of *FLX* was cloned into pQH05 vector with a HIS3 selection marker. Various combinations of AD and BD vectors with pQH05 or pQH05-FLX were co-transformed into Y2HGold yeast and grown on SD-His/-Leu/-Trp and SD-Ade/-His/-Leu/-Trp mediums.
